# Flumazenil; a Medication Considered for Reversal of Benzodiazepine Overdose in Rapid Tranquillisation, but Could Administering It Be Hazardous for Both Patient and Doctor? A Rapid Review Required

**DOI:** 10.1192/bjo.2025.10281

**Published:** 2025-06-20

**Authors:** Rebecca Hutchinson

**Affiliations:** NIMDTA, Belfast, United Kingdom

## Abstract

**Aims:** Flumazenil is a benzodiazepine antagonist and competitively inhibits binding at the benzodiazepine receptor. In Northern Ireland (NI) mental health hospitals, and across other UK mental health hospitals, flumazenil is documented as the reversal agent for use in iatrogenic benzodiazepine overdose (usually lorazepam), as part of the rapid tranquillisation protocol. The indications for its use, contraindications and risks of use need to be considered carefully for safe and appropriate prescribing.

**Methods:** **Case report (or lack thereof):** Within the recent past, flumazenil has not been prescribed or administered to any patient receiving rapid tranquillisation within the Northern Trust mental health service in NI. The following is a hypothetical but common situation for consideration: A detained patient (under The Mental Health Order Northern Ireland 1986) presents with acute aggression/distress due to first episode drug-induced psychosis. They have no past medical or psychiatric history and are not on regular medication. They have not responded to other less restrictive interventions and the decision has been made to prescribe first-line rapid tranquillisation, lorazepam 1mg IM. Following this, the patient is noted to become sedated with a respiratory rate of 10 and oxygen saturations of 94%. The current policy suggests prescribing flumazenil.

**Results:** On review of multiple resources, including British Association for Psychopharmacology Guidelines, The Maudsley Prescribing Guidelines in Psychiatry 14th Edition, British National Formulary, Electronic Medicines Compendium, Toxbase and local rapid tranquillisation policies throughout the UK, there appears to be a discrepancy in the advice given on the circumstances in which it would be safe to prescribe flumazenil. Some resources document that flumazenil can be considered in iatrogenic benzodiazepine overdose in patients who do not have long term benzodiazepine use or mixed intoxications, with others noting that it should be given by an “experienced” doctor. When flumazenil is used in a physical health setting its use is mostly limited to intensive care/post surgery, and should be given by a doctor with experience in anaesthesiology. Convulsions and cardiac arrhythmias are serious adverse effects of flumazenil use.

**Conclusion:** The use of flumazenil following iatrogenic benzodiazepine overdose is rare in the Northern Trust, NI, however, as it is available on all mental health wards it is paramount to highlight the importance of careful selection of patients, in which it may be indicated and beneficial, and the risks surroundings its use. The limited experience of most psychiatric doctors in using this drug would suggest it should not be administered in a stand-alone mental health unit.

